# Transverse process strut and titanium mesh cages in the stability reconstruction of thoracic single segment tuberculosis: a retrospective single-center cohort study

**DOI:** 10.1186/s12891-020-03196-3

**Published:** 2020-03-16

**Authors:** Weiyang Zhong, Xinjie Liang, Ke Tang, Xiaoji Luo, Zhengxue Quan

**Affiliations:** 1 Department of Orthopedic Surgery, The First Affiliated Hospital of Chongqing Medical University, Chongqing, China; 2 Department of Pain Management, The First Affiliated Hospital of Chongqing Medical University, Chongqing, China

**Keywords:** Transverse process strut, titanium mesh cages, Thoracic spinal tuberculosis

## Abstract

**Background:**

A retrospective and comparative study of transverse process strut (TPS, Group A) compared with titanium mesh cages (TMCs, Group B) in the reconstruction of thoracic stability through the one-stage posterior approach to treat single-segment tuberculosis.

**Methods:**

Sixty patients from January 2013 to December 2016 were analyzed and divided into two groups. The following data of clinical and radiographical assessments were observed preoperatively, postoperatively and during follow-up (FU).

**Results:**

The patients were followed up for an average of 50.20 ± 25.10 months (Group A) and 48.70 ± 27.30 months(Group B) without significant difference. No significant differences were found in the mean of operation time in minutes, blood loss, hospitalization time, drainage and follow-up duration between the groups. The VAS, ODI, ESR and CRP were reduced significantly at the final FU compared with the preoperation values and there was no significance between the groups. Neurological deficits were improved in all patients at the final FU without significant difference between the groups(*P* > 0.05). The bony fusion times were 5.85 ± 1.82 months and 8.4 ± 5.1 months with significant difference(*P* < 0.05). Comparing with the preoperative values, the kyphosis angle significantly improved, but at the final FU the significant difference was found between the groups (*P* < 0.05). The loss of the angular correction and the fused segmental height in group A was lower than that in group B (*P* < 0.05).

**Conclusions:**

TPS had a better osseous fusion rate, effective maintenance of fused segment stability which is a good bone graft for surgical management of single-segment thoracic spinal tuberculosis.

## Background

Human tuberculosis (TB) is a chronic and ancient disease characterized by weight loss and it is a cause of death and most cases of tuberculosis occur in developing countries. According to the World Health Organization (WHO), TB causes 1.81 million deaths in Asia each year and China has 78% new cases every year [1–4]. In recent years, the emergence of Acquired Immune Deficiency Syndrome (AIDS) and drug resistance has greatly increased the incidence of tuberculosis and made it a bigger threat, arousing considerable attention worldwide [5, 6]. The spine is involved in 50% of osteoarticular tuberculosis cases, spinal involvement is especially dangerous, as it can result in destruction of the vertebral body, spinal deformity and/or paraplegia. When the affected spine becomes unstable, the resulting deformity creates a risk of spinal cord compression. The goals of tuberculosis treatment are to eradicate the infection provide spinal stability and save the patient’s life. Supportive care, chemotherapy and surgery are consistently effective in the management of spinal tuberculosis [7, 8].

When severe bone destruction, kyphosis, or nerve deficit occurs, surgical management still plays a significant role in curing spine tuberculosis. After debridement and decompression, many interbody bone grafts are used for to reconstruct spinal stability, such as the iliac crest which is the gold standard or fibula grafts and titanium mesh cages (TMCs) [9, 10]. Autologous bone or allografts can achieve good bony fusion, however, they have some problems that limit their application. A number of studies have reported that TMCs shows significant potential for reliable spinal reconstruction and it offers excellent bone fusion, adequate sagittal profile maintenance and a low implant-related complications rate [11–15]. In our study, the transverse process strut (TPS) was used as a bone graft to reconstruct the stability of the spine. The purpose of the research was to investigate the clinical effect of TPS compared with TMCs for the surgical treatment of single-segment thoracic spinal tuberculosis.

## Methods

### Patients

This study was approved by the Institutional Review Board of The First Affiliated Hospital of Chongqing Medical University. Two treatment plans were involved in the routine clinical care. When communicating with patients before surgery, the advantages and disadvantages of the two types of management were fully introduced, so the patients could choose the right treatment for themselves. All surgical procedures were performed by the same senior surgeon.

From January 2013 to December 2016, 60 patients with single-segment thoracic spinal tuberculosis were treated and reviewed retrospectively in our department. In group A, 30 patients received TPS for the reconstruction and in group B, 30 patients underwent TMCs. Inclusion criteria: adult single-segment thoracic spinal tuberculosis who were indicated for surgery: increasing kyphosis, neurological deficits, bone destruction affecting the stability, the bone damage of the infected vertebral body did not exceed 1/2 the vertebral height, one-stage posterior approach for internal fixation, and reconstruction. Exclusion criteria: active tuberculosis of the lung and extrapulmonary organs, multiple-segment thoracic vertebral tuberculosis, cancer.

Chemotherapy with an HREZ standard chemotherapy regimen comprising isoniazid(H)(5-10 mg/kg/day), rifampicin(R)(10 mg/kg/day), ethambutol(E)(15 mg/kg/day), and pyrazinamide(Z)(25 mg/kg/day) was administered 3-4 weeks before the operation. Surgery was performed when the patient’s erythrocyte sedimentation rate (ESR), C-reactive protein (CRP) and temperature recovered to normal or nearly normal. If the patient’s neurological condition deteriorated rapidly, an urgent salvage operation was performed.

### Surgical technique and measurement

**Fig. 1:**
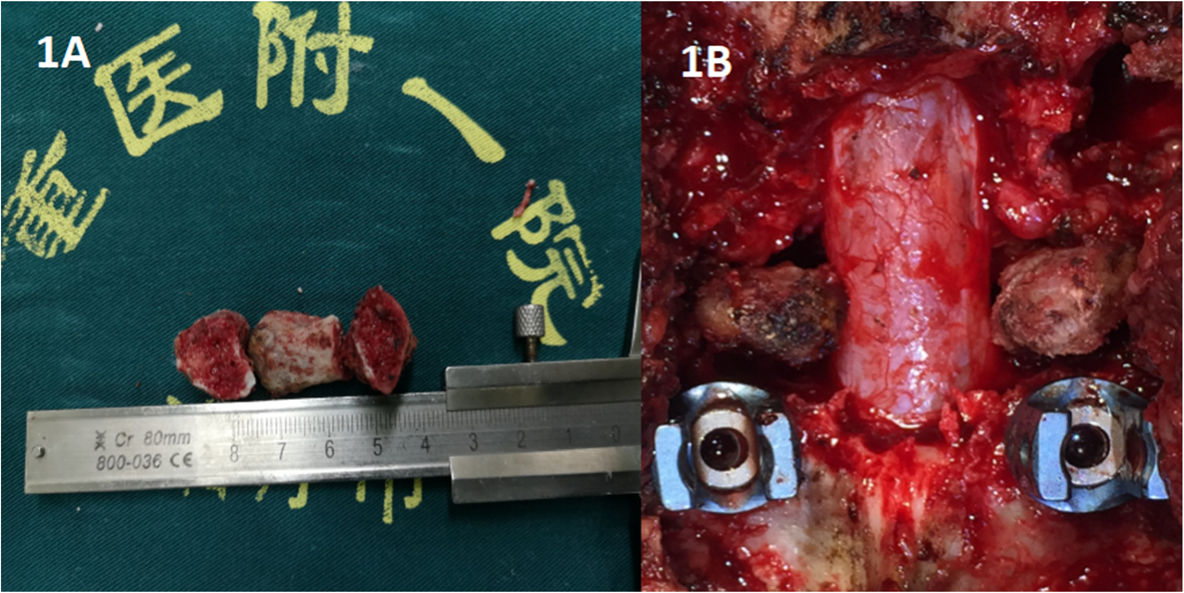
Photographs of one, two or three trimmed transverse process that were implanted

In group A, the patient was placed in prone position after the administration of conventional tracheal anaesthesia. The posterior elements, including the lamina, facet joint and transverse process, were exposed with a midline incision. Pedicle screws were fixed on the basis of imaging and C-arm X-rays to ensure their accuracy. After decompression and thorough debridement, the adjacent transverse process was cut off and trimmed to round cortical bone and double-sided cancellous bone (Fig. 1). Depending on the characteristics of the space after thorough debridement, one or more TPS was implanted in the anterior defect and the device was properly locked. Topical streptomycin 1.0 g and isoniazid 0.2 g mixed gel were applied. After surgery, negative pressure drainage was used to remove specimens and conduct bacterial cultures and pathology examinations. In group B, the surgical procedure was similar to that described for group A. The only difference was that the trimmed TMCs (filled with removed cancellous bone) was implanted into the bone defect and fully connected to the upper and lower vertebral end-plates.

The drainage tube was removed when less 50 ml/day was drained from the surgical site. The patients received oral HREZ chemotherapy after surgery for 12 months, followed by 6-month regimens of HRE chemotherapy (12HREZ/12-18HRE). Rehabilitation training and nutritional support began the day after surgery. Ambulation with a brace was allowed the third day after surgery. All patients were evaluated clinically and radiologically at one week and 3, 6 and 12 months after surgery and once a year thereafter.

**Fig. 2:**
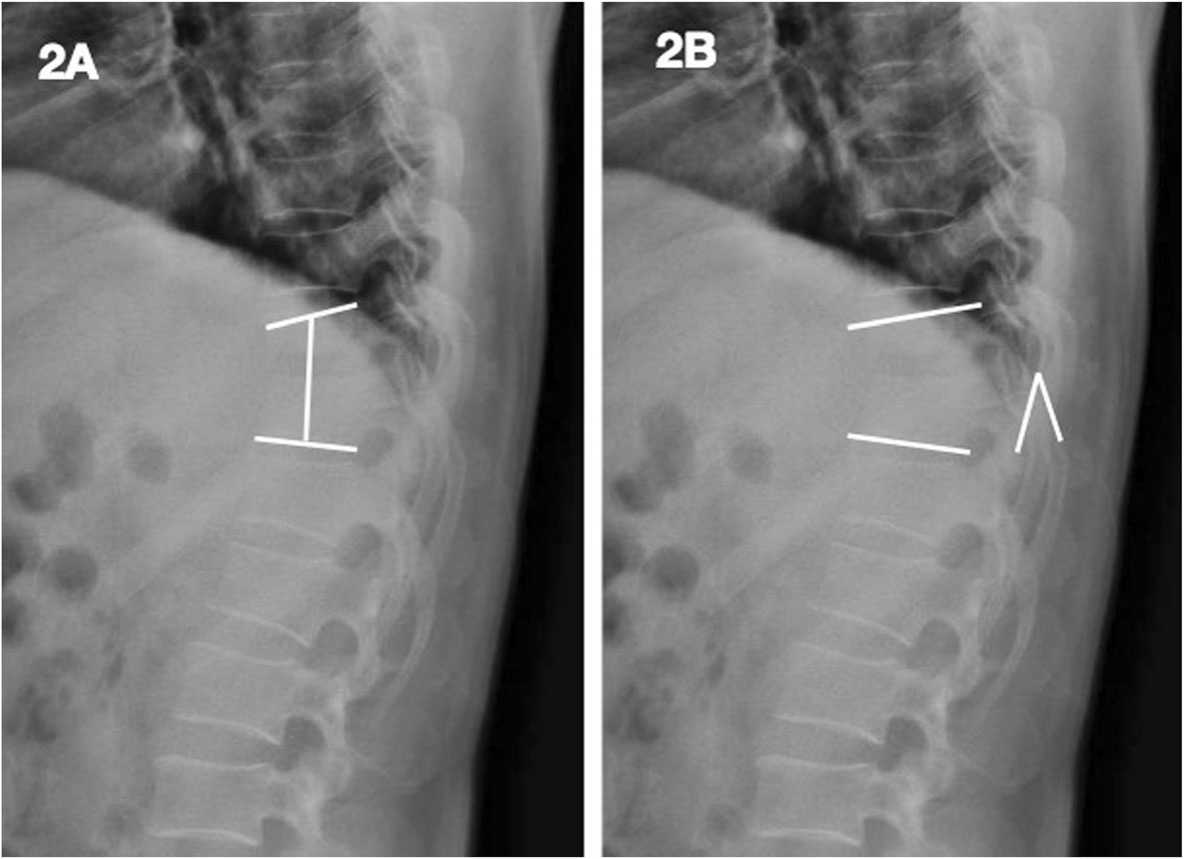
Presentation of the measurement of the fused segmental height (**a**) and of the kyphosis angle (**b**)

For all patients, the following data were observed preoperatively, postoperatively, and during follow-up (FU): (1) the operation time, surgical blood loss, hospitalization time, drainage, bone fusion time (bone graft fusion was assessed using the radiologic criteria of Bridwell et al. [15], 2) kyphosis angle; neurological function (American Spinal Injury Association, ASIA); (4) Visual Analogue Scale (VAS) and Oswestry Disability Index (ODI); (5) erythrocyte sedimentation rate (ESR) and C-reactive protein (CRP). The fused segmental height (the distance between the midpoint of the superior endplate of the upper vertebral body and the midpoint of the inferior endplate of the lower vertebral body)) was measured (Fig. 2). The height loss was calculated with the following formula: (Height of 1 week post-operation) − (Height of subsequent follow-up).

According to the Cobb method, the kyphosis angle was defined as the angle formed between the superior endplate of the upper vertebral body and the inferior endplate of the lower vertebral body (Fig. 2). Loss of angular correction was calculated with the following formula: (Angle of 1 week post-operation) − (Angle of subsequent follow-up). The subsidence was defined as a decrease in the fused segmental height during the follow-up period greater than 3 mm compared with that on day 1. All radiographic data and measurements in this study were reviewed by one senior spine surgeon and one senior radiologist.

### Statistics

All statistic data were analysed with SPSS version 22.0 statistical soft ware (SPSS, Inc., Chicago, IL, USA). Quantitative data are presented as means and standard deviations. Repeated measures t-test was used for the statistical analysis of differences in mean values, and the chi-squared test was used for categorical data. Statistical significance was defined as a *p* value < 0.05.

## Results

The patients were followed up for an average of 50.20 ± 25.10 months (group A) and 48.70 ± 27.30 months (group B) without significant difference. No significant differences were found in the mean of operation time in minutes, blood loss, hospitalization time,drainage and follow-up duration between the groups(*P* > 0.05) (Table 1). The VAS, ODI, ESR and CRP were reduced significantly at the final FU compared with the preoperation values and there was no significant significance between the groups. Neurological deficits were notably improved in all patients at the final FU without significant difference between the groups(*P* > 0.05) (Table 2).

**Table 1:** Comparison of the baseline and postoperative data

	Group A	Group B	*P* value
No. of patients (*n*)	30	30	
Male/female (*n*)	21/9	16/14	
Mean age (years)	46.23 ± 17.20	45.78 ± 19.10	0.912
Hospital stay (days)	17.31 ± 4.23	18.50 ± 2.88	0.874
Surgery time (minutes)	195.08 ± 24.07	190.30 ± 26.15	0.780
Blood loss (ml)	280.77 ± 189.90	300.77 ± 150.60	0.437
Drainage (ml)	436.92 ± 193.81	402.92 ± 169.21	0.356
Mean fusion time (months)	5.85 ± 1.82	8.4 ± 5.1	0.012
Follow-up (months)	50.20 ± 25.10	48.70 ± 27.30	0.650

**Table 2:** Clinical and radiographic outcomes in each group

Parameter	Group A	Group B	P value
ESR (mm/h) change from before treatment to final follow-up	51.31 ± 18.54	58.12 ± 15.32	0.125
	10.61 ± 2.96*	12.52 ± 1.25*	0.223
VAS score change from before treatment to final follow-up	6.4 ± 0.88	6.7 ± 0.45	0.654
	1.62 ± 0.65*	1.71 ± 0.35*	0.699
ODI score change from before treatment to final follow-up	39.15 ± 4.02	40.05 ± 3.22	0.774
	4.68 ± 1.43*	4.83 ± 1.15*	0.805
Severity of neurological deficits			
Before treatment			
ASIA A			
ASIA B			
ASIA C	18	19	0.721
ASIA D	10	8	0.735
ASIA E	2	3	0.810
At the final follow-up			
ASIA A			
ASIA B			
ASIA C			
ASIA D	2	1	0.794
ASIA E	28	29	0.808
Kyphosis angle(^。^) before treatment the final follow-up	18.77 ± 2.49	17.97 ± 2.80	0.301
	9.31 ± 1.54*	6.25 ± 1.04*	0.009
Loss of angular correction (^。^)			
1 week post-op	1.10 ± 1.10	1.25 ± 1.05	0.701
3 months post-op	6.50 ± 3.50	6.90 ± 4.30	0.030
6 months post-op	7.70 ± 3.30	9.50 ± 4.05	0.024
12 months post-op	8.95 ± 4.60	11.55 ± 4.85	0.012
Final follow-up	9.60 ± 1.12	11.85 ± 1.80	0.006
Height loss of fused segment (mm)			
1 month post-op	0.68 ± 0.23	0.58 ± 0.33	0.853
3 months post-op	1.07 ± 0.66	1.94 ± 0.51	0.021
6 months post-op	1.90 ± 0.74	2.48 ± 1.36	0.015
12 months post-op	2.08 ± 0.85	2.64 ± 1.42	0.010
Final follow-up	2.58 ± 0.45	2.78 ± 0.95	0.007
Implant subsidence	3.3%(1/30)	13.3%(4/30)	0.122

*P < 0.05: at the final follow-up vs. before treatment

*VAS* visual analogue scale, *ODI* Oswestry Disability Index, *ESR* erythrocyte sedimentation rate, *CRP* C-reactive protein, *ASIA* American Spinal Injury Association

**Fig. 3:**
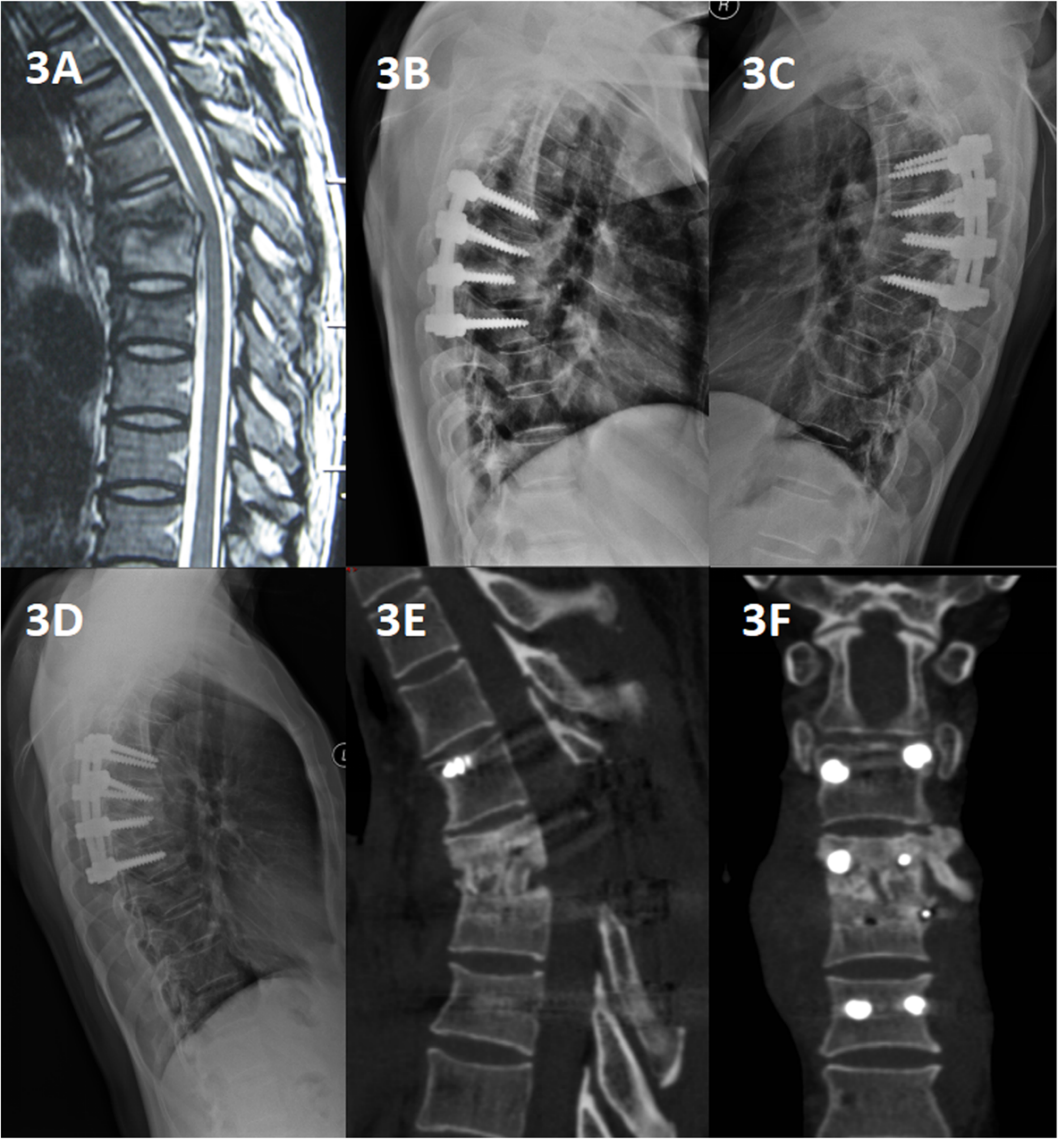
An 18-year-old man with thoracic spinal tuberculosis (T6-7) underwentsingle-segment posterior debridement and decompression combined with internal fixation. (**a**) Pretreatment MRI showing destruction of the T6-7 vertebrae and concomitant compression of the spinal cord. (**b**,**c**) Immediate postoperative and 6-months radiographs demonstrating posterior debridement, bone graft and internal fixation. (**d**,**e**,**f**) At the 36-month follow-up, plain Xray and CT showed maintenance of the correction and solid fusion

**Fig. 4:**
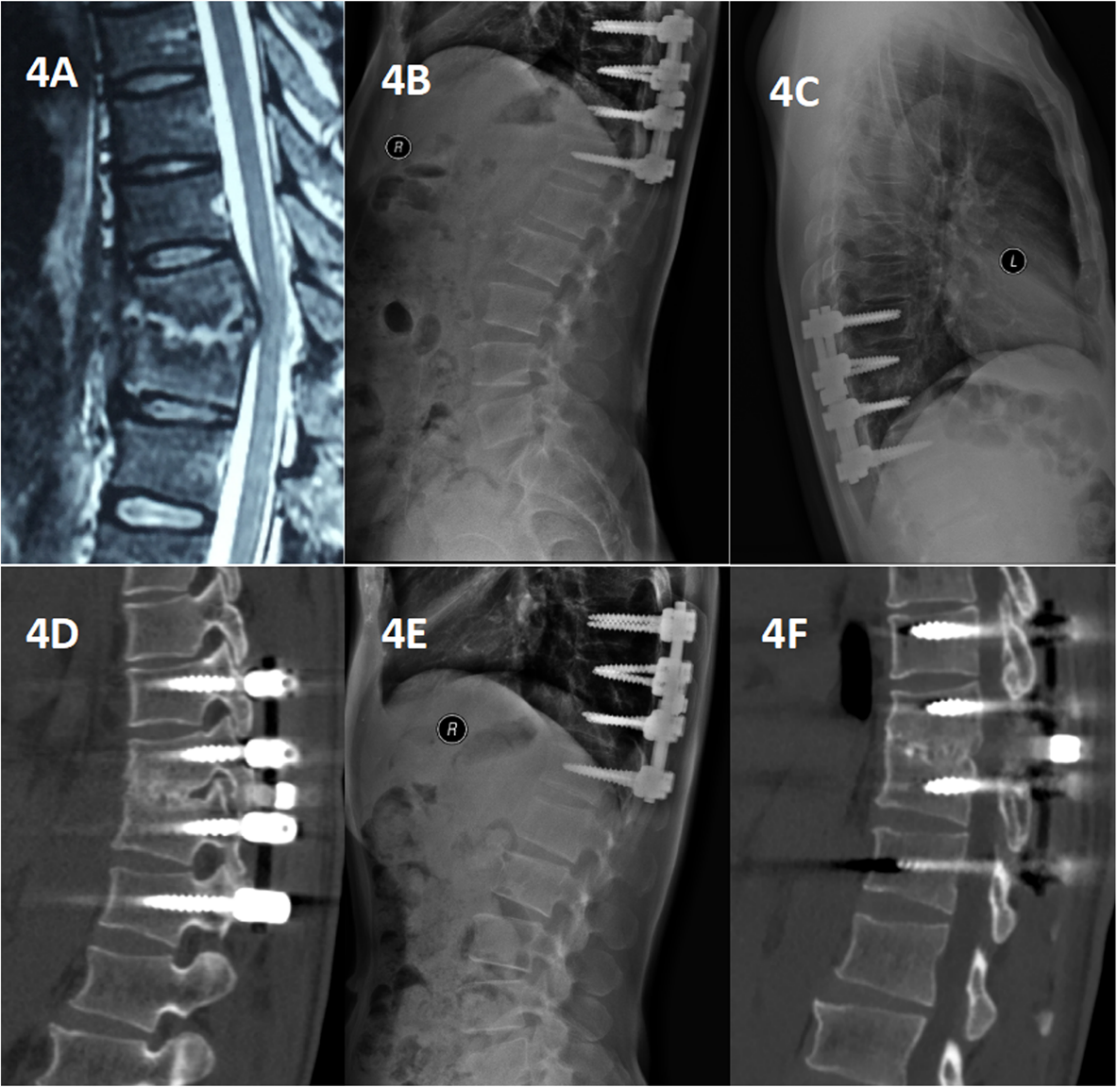
A 40-year-old man with thoracic spinal tuberculosis (T10-11) underwent posterior debridement and decompression combined with internal fixation. (**a**) Pretreatment MRI showing destruction of the T10-11 vertebrae and concomitant compression of the spinal cord. (**b**,**c**,**d**) Immediate postoperative and 6-month radiographs and 6-month CT demonstrating posterior debridement, bone graft and internal fixation. (**e**, **f**) At the 22-month follow-up, plain X-ray and CT showed maintenance of the correction and solid fusion

**Fig. 5:**
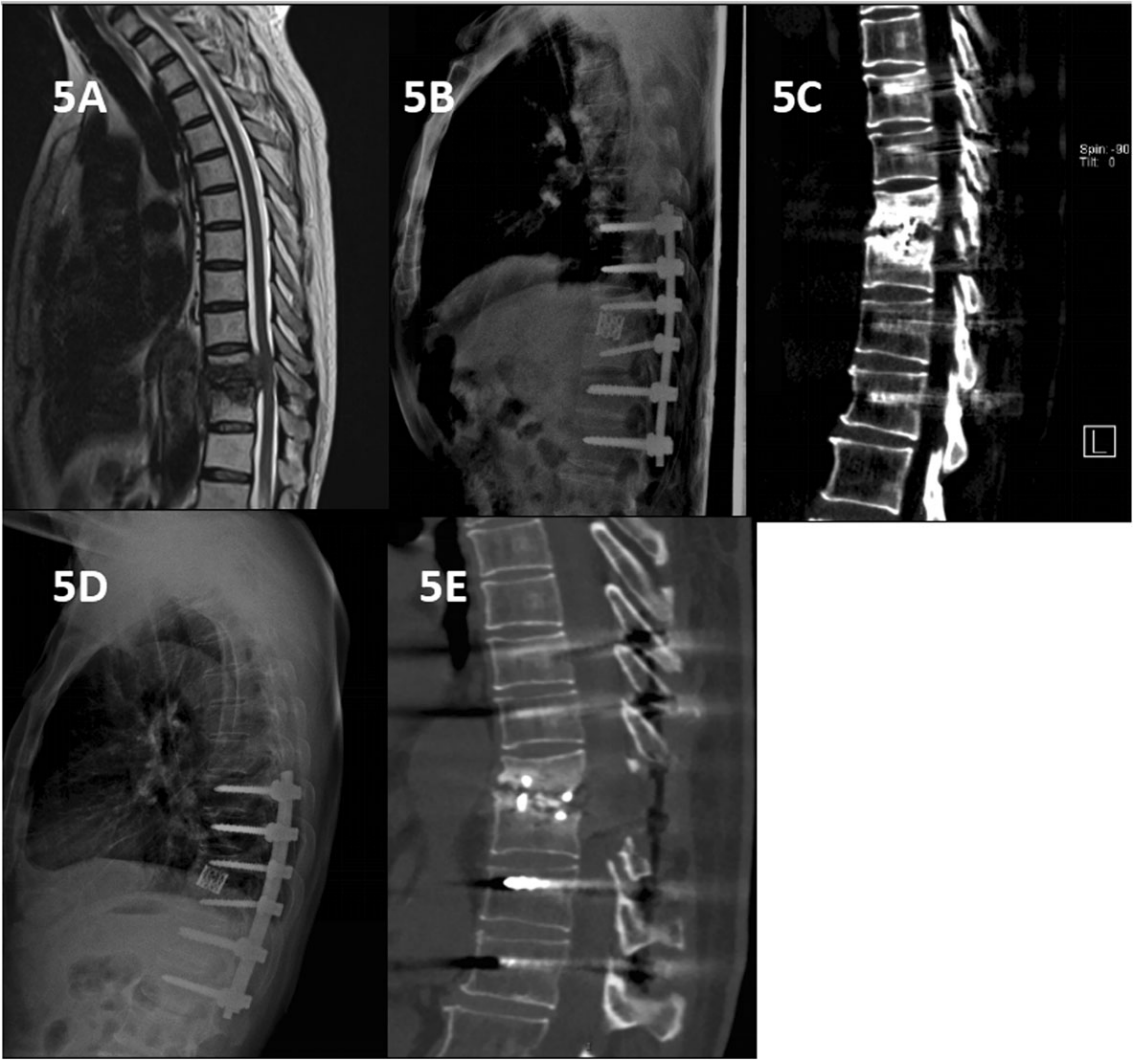
A 42-year-old man with thoracic spinal tuberculosis (T11–12) underwent posterior debridement and decompression combined with internal fixation. **a** Pre-treatment MRI showing the destruction of the T11–12 vertebrae and concomitant compression of the spinal cord. **b** Immediate postoperative radiographs demonstrating posterior debridement, bone graft and internal fixation. **c, d, e** At the 24-month follow-up, plain X-ray and CT showed maintenance of the correction and solid fusion, good clinical effect and subsidence of the titanium mesh cage

Spinal TB was completely cured and all patients achieved bone fusion. The bone fusion time was 5.85 ± 1.82 months in group A and 8.4 ± 5.1 months in group B and the difference was significant. Comparing with the preoperative values, the kyphosis angle significantly improved, but at the final FU, the significant difference was found between the groups (*P* < 0.05). The loss of angular correction and fused segmental height in group A was lower than that in group B (*P* < 0.05) (Figs. 3, 4, and 5).

No complications, including bone graft failure, pleural effusion and wound infection, were recorded, with the exception of cerebrospinal fluid leakage (one case in group B), fistula (one case in group A), water electrolyte imbalance (three cases in group A, five cases in group B), superficial infection (one case in group B) and mild intestinal obstruction (one case in group A, one case in group B). Implant subsidence occurred in five cases (one case in group A, four cases in group B) and the difference between groups was not significant.

## Discussion

The TB incidence is rising worldwide with 9 million new cases and 2 million deaths per year caused by the increased incidence of AIDS and resistance to anti-tuberculosis drugs [1–6]. Spinal TB remains a common spinal infection. Due to vertebral body collapse or severe deformity, surgical options may be considered appealing in conjunction with the more common conservative treatment options, such as anti-tuberculosis drugs, which are vital. Formal, appropriate and long-term anti-tuberculosis chemotherapy, strict bed rest, and supportive treatment are essential therapies [7–12]. Despite the availability of effective conservative treatments, the goals of current surgical management for spinal TB include debridement of the infected vertebrae, decompression of the spinal cord, correction of deformities, stabilization of the spine and further protection of the spinal cord. Radical debridement and corrective and stabilizing spinal surgery is most effective in cases of active, progressive kyphosis and nonrigid kyphosis [13–15]. The anterior surgery has several disadvantages such as significant blood loss, a longer operation time, and more severe complications and it’s more challenging comparing with posterior surgery [16, 17]. Hence, the therapeutic strategy for this disease has been modified in recent years to become more accurate and individualized.

After debridement and decompression, many types of interbody bone grafts may be used to reconstruct the anterior and middle column. Autogenous bone graft, such as an iliac crest or fibula graft, can achieve good bony fusion, however, they can lead to complications at the donor site, such as pain, blood loss, hematoma and unhealed wounds. Allografts have been used to avoid donor site complications, but they are associate with decreased arthrodesis rates and increased graft collapse rates. Recently, a number of studies have reported that TMCs showed significant potential for reliable spinal reconstruction, offering excellent bone fusion, adequate sagittal profile maintenance and a low rate of implant-related complications. In our study, TMCs were filled with cancellous bone from excised vertebral lamina and articular processes, which allowed their design to be individualized according to different bone defects [18, 19]. Furthermore, donor site complications did not occur. In addition, their implantation in the defect provided an adequate bony interface to assure spinal stability. The current clinical outcomes showed that solid bony fusion, a satisfactory clinical effect and improved neurological function were obtained in the single-segment titanium mesh group and the transverse process strut group. Although some cases subsided, spinal alignment and stability were still maintained [20].

Cui et al. reported that the thoracic transverse process was longer, thicker, higher and larger than the transverse processes of vertebrae at other spinal levels and could be used as a bone graft. Maria E. K.et al. and other studies demonstrated the quantitative three-dimensional anatomy of the thoracic vertebrae and the discs. Given these anatomical features, the transverse process satisfies the needs of grafts if the bone damage of the infected vertebral body did not exceed 1/2 the vertebral height [21, 22]. The use of the transverse process as a bone graft strut can avoid donor site complications. The transverse process, as an autogenous bone, can be trimmed to the specifications of the bone defect,which not only reduces the difficulty of implantation but also ensures the strength of bone grafting and creates a large interface between the bone graft and the end plate, assuring good fusion. Compared with TMCs [23–28], TPS has superior biomechanical features and multiple transverse bone grafts can be used if the defect requires them. In our study, we used TPS combined with internal fixation; all patients showed satisfactory fusion, and the postoperative kyphosis angle was significantly corrected, indicating that TPS can effectively support the spine. Compared to TMCs, TPS can achieve good clinical outcomes and it presented a significantly earlier bony fusion time. As a bone implant, the strength and fusion properties of TPS can effectively reconstruct and maintain spinal stability clinically. Furthermore, the use of TPS can reduce the hospital costs.

## Conclusion

The results of this study showed that when used as a bone graft reconstruct the stability of single-segment thoracic tuberculosis, TPS has a better rate of osseous fusion than TMCs and can provide effective restoration and maintenance of fused segment height and alignment and a lower rate of complications. TPS is an ideal reconstructed bone graft for thoracic single segmental stability. However, we declare that there were a few limitations in the study. First, the study didn’t consider the intra and inter observer differences which was associated with bias. Second, the retrospective nature of the small-sample study may be associated with bias. Third, if the bone damage of the infected vertebral body did exceed 1/2 the vertebral height, TPS was not long and strong enough to stabilize which could be prone to subside, or even result in kyphosis and the TMCs or iliac bone graft could be more suitable. Fourth, we need to perform the biomechanical tests between the groups to know the difference of the strength, which could confirm that TPS meets the mechanics needs. In the future, the prospective, randomized studies with long-term follow-up periods are needed.

## Data Availability

The datasets generated and/or analysed during the current study are not publicly available due to the data is confidential patient data but are available from the corresponding author on reasonable request.

## Abbreviations

TB: Tuberculosis
TPS: Transverse process strut
TMCs: Titanium mesh cages
WHO: World Health Organization
AIDS: Acquired Immune Deficiency Syndrome
ASIA: American Spinal Injury Association
VAS: Visual Analogue Scale
ODI: Oswestry Disability Index
ESR: Erythrocyte sedimentation rate
CRP: C-reactive protein
FU: Follow-up
